# Performance evaluation of BD FACSPresto^™^ point of care CD4 analyzer to enumerate CD4 counts for monitoring HIV infected individuals in Nigeria

**DOI:** 10.1371/journal.pone.0178037

**Published:** 2017-05-25

**Authors:** Olubunmi Ruth Negedu-Momoh, Feyisayo Ebenezer Jegede, Ali Yakubu, Oluseyi Balogun, Musa Abdullahi, Titilope Badru, Edward Adekola Oladele, Chinedu Agbakwuru, Hadiza Khamofu, Kwasi Torpey

**Affiliations:** 1 Family Health International (FHI360), Garki- Abuja, Nigeria; 2 Murtala Mohammed Specialist Hospital, Kano, Nigeria; 3 University of Ghana College of Health Sciences, Accra, Ghana; Universita degli Studi di Roma Tor Vergata, ITALY

## Abstract

**Background:**

Despite the upsurge in support and intervention of donor agencies in HIV care and treatment programing in Sub-Sahara African, antiretroviral (ART) programs are still confronted with access and coverage challenges which influence enrolment of new patients. This study investigated the validity of point of care BD FACSPresto^™^ CD4 analyzer for CD4+ cell count, overall agreement, correlation, sensitivity, and specificity in comparison to a reference standard flow cytometry method. We also assessed the feasibility of use among non-laboratorians.

**Methods:**

Blood samples from 300 HIV infected individuals were analyzed for CD4+ T cell and CD4%, using finger prick capillary sample from 150 PMTCT clients and 150 ART clients at Murtala Mohammed Specialist Hospital, Kano, Nigeria. Their venous samples were compared on a flow cytometry reference method using BD FACSCount CD4+ count system. The accuracy of the BD FACSPresto machine in comparison to BD FACSCount was evaluated. Statistical analysis was carried out using STATA (version 12). Bland-Altman method and correlation analysis were used to analyze agreement between both measurements. In addition, sensitivity and specificity of both measurements were determined. Statistical significance was set at p-value <0.05.

**Results:**

The mean bias and limit of agreement for CD4+ count between BD FACSPresto and BD FACS count machine were 7.49 (95% CI: 2.44 to 12.54) and -8.14 to 96.39 respectively. Further analysis revealed close agreement between BD FACSPresto and BD FACSCount with no significant difference between the two methods (p = .0.95). Using a threshold of 500 cells/μL, sensitivity and specificity of BD FACSPresto were 95.1% and 97.1% respectively, compared to BD FACSCount. There was no statistically significant difference in the misclassification between BD FACSPresto and BD FACSCount results (p = 0.23). Furthermore, sensitivity and specificity were similar when BD FACSPresto machine was operated by a nurse or laboratory scientist, there was no substantial difference in testing variability observed between laboratory and non-laboratory operators using the BD FACSPresto analyzer.

**Conclusions:**

Overall, BD FACSPresto Point of Care CD4+ count finger stick capillary blood results is a reliable method in comparison to venous sample cytometry method and no significant difference variability observed between laboratory personnel and non-laboratory operators. The BD FACSPresto is simple, more robust and easy to use equipment without significant variability in reliability by non-laboratory health care workers hence will be a valuable instrument in increasing access and coverage of CD4 estimations in developing countries. The introduction of the BD FACSPresto POC analyzer has a high potential in reducing patients waiting time and improving the overall quality of ART service and clients’ satisfaction especially in rural settings.

## Introduction

HIV is chronic infection that requires periodic monitoring for proper management and care. CD4 estimation is one of the methods for client monitoring on ART. CD4 estimation had played a role in treatment initiation previously but the 2016 WHO guidelines recommended that all HIV infected persons be initiated on ART regardless of WHO clinical stage or CD4 cell count [1]. Within the context of these guidelines, CD4 estimation continues to play a role in treatment prioritization and client monitoring. The WHO guideline and recommendations were adopted by Federal Government of Nigeria in 2016 and are now in place as the national ART guideline [2]. Viral load testing which is now placed as the as a key tool for monitoring adherence to treatment and disease progression [3] is however only just being scaled up. Many resource limited countries are confronted with many challenges ranging from capacity and infrastructure to meet up with the demand of viral load testing in resource limited settings like Nigeria [4]. Latest literature revealed that cost and complexity including clinicians awareness have been identified as limitation to scale-up of viral load testing in developing countries [5].

Therefore, CD4 count continues to play an important role in measuring baseline immune compromise, and prioritizing decisions regarding ART initiation in settings where universal treatment is not available. In many resource limited settings, immunological measurement serves as an important marker for monitoring treatment failure and a guide for routine follow up care where PCR laboratories are not available to monitor patient viral load [5, 6]. Baseline CD4 determination is alsobe useful and relevant in determining patients who requiring screening for cryptococcal infection and subsequent prophylaxis. However, state-of-the-art CD4+ T cell counting methods based on flow cytometry principle and technique are not readily available especially in rural settings due to their high cost and technical requirements [7].

In the developing world, CD4+ T cell tests are often performed typically in centralized laboratories in urban settings with limited access to such services in the rural areas; clients receiving services at the lower level health centers such as Primary Health care centers can only access CD4+ T testing through sample transfer to some centralized laboratories for analysis or by referral of the clients themselves in few cases. Patients have to make multiple visits to central health facilities with laboratories to obtain a single test result and this becomes a barrier for expansion and decentralization of ART programme and adherence to treatment [1].

Despite support and intervention of donor agencies on ART programming, sub-Sahara African ART program is still confronted with challenges; such as fewer facilities with capacity to perform CD4+ T cell count, frequent breakdown of CD4+ T cell count machines, non-availability of reagents, degraded reagent due to poor storage and erratic power supply thus causing delays in obtaining test results, long patient’s backlogs and increase in patients waiting periods between tests and, subsequently, delays in the initiation of ART or prophylaxis [8]. It had been documented that loss to follow-up of HIV-positive patients before initiation of antiretroviral therapy can exceed 50% in low-income settings and is a challenge to the scale-up of ART [9]. Consequently, the need for expansion of ART programs into rural areas has created a need for faster CD4 counting alternatives with limited technicalities [10]. Point of care (POC) CD4 enumerating equipment can make the CD4 count available at sites with reduced turnaround time and improved access to ART services, thereby improving patients' management considerably [9, 11]. Therefore, POC CD4 equipment was designed to be able to provide results within a relatively shorter time compared to standard equipment.

Accurate, inexpensive POC CD4+ T cell testing technologies are needed with ability to deliver CD4+ T cell results at lower level health centers or community outreach voluntary counseling and testing or in a stand-alone prevention of mother to child transmission (PMTCT) settings [6]. Additionally, POC CD4 testing may play an important role in identifying individuals who require antiretroviral therapy (ART) [12]. To address challenges associated with conventional CD4 count testing, World Health Organization in 2013 (WHO) released consolidated ART guideline which reflects on advances in HIV responses and the use of new technologies like CD4 point-of-care and new service delivery approaches in allowing HIV testing and treatment monitoring to be diversified and decentralized [8].

BD FACSPresto is a new point-of-care system developed by BD Biosciences in San Jose, California in 2014 with an integrated software to provide absolute CD4^+^ cell counts and CD4% count for staging and monitoring HIV patients and total hemoglobin (Hb) concentration of whole blood samples in single preparation. BD FACSPresto cartridge kit uses fluorochrome-conjugated antibody reagents and integrated reagent quality control. BD FACSPresto consists of fluorescence imaging and absorbance reading technology, with embedded software to analyze patient samples from a single-use disposable cartridge. BD FACSPresto POC system CD4 testing technologies is small and portable bench-top POC system operated using 100–240 V (AC) power source and designed to fit the need of a remote location using a rechargeable battery system. It can measure absolute CD4 counts, percent CD4 and hemoglobin from a single drop of capillary or venous blood in approximately 23 minutes with through put of 10 samples per hour [13].

Validation of POC testing with other equipment such as PIMA has been done [11, 14–16]. BD FACSPresto has also been evaluated in South Africa [17], and Kenya [13, 18] but there is no previous evaluation of the performance of BD FACSPresto system in Nigeria. Moreover, due to the increasing number of new diagnostic tests and point-of-care systems entering the diagnostic market, WHO 2013 consolidated guidelines recommended the need for high quality diagnostics and equipment with strategic planning on placing and harmonizing testing platforms to ensure appropriate use and cost–effectiveness [8]. Thus, for accurate and reliable CD4 results, validation of these platforms under program conditions is essential. The objective of this study was to investigate the validity of point of care BD FACSPresto^™^ CD4 analyzer for CD4+ cell count, overall agreement, correlation, sensitivity, and specificity in comparison to a reference standard flow cytometry method. We also assessed the feasibility of use among non-laboratorians.

## Methods

### Study site

The study was conducted at Murtala Mohammed Specialist Hospital (MMSH), Kano, one of the secondary hospitals located in Kano state, North-western Nigeria. The site has been supported by FHI 360 (Family Health International) to provide comprehensive ART program since June 2006 till date with funding from President’s Emergency Plan for AIDS Relief (PEPFAR) through United States Agency for International Development (USAID). The site has an average of over 6,000 out-patients recorded daily. It is the largest hospital owned by the Kano State Government (State Ministry of Health) and has the highest client volume. Of the 18 ART facilities that met the inclusion criteria, MMSH was purposively selected. In this facility, patients who present for the first time are offered HIV testing and counseling. Those that test HIV positive are enrolled into the ART program. The PMTCT services is integrated into antenatal clinic (ANC) and maternal child health (MCH) services. Women testing HIV positive are enrolled into the PMTCT program.

The laboratory supports HIV screening and disease monitoring tests such as CD4+ enumeration, toxicity and opportunistic Infections (OI) assays. The laboratory has BD FACSCount analyzer (Beckton Dickinson, USA) which was used as the reference method in this study. The instrument is registered for the external proficiency assessment scheme of National Health Laboratories (NHLS) South Africa. The BD FACSCount analyzer at MMSH uses CD4 absolute and CD4% count floppy diskette software. Prior to this study, two laboratory scientists and a nurse from the PMTCT clinic were trained for 2 days on CD4 count estimation using BD FACSPresto analyzer, finger prick and venous sample collection and biosafety. The BD FACSPresto^™^ analyzer and reagents were provided by BD Bioscience USA.

### Study participants

Study participants were enrolled between 30^th^ September 2015 to March 2016. Informed, consenting patients 18 years and above who tested positive for HIV infection, attending MMSH ART clinic and pregnant women 18 years and above, currently attending PMTCT clinic were considered eligible for the study. A total of 300 HIV infected patients were recruited and relevant bio data information collected. Patients who did not sign consent form were disallowed from participating in the study but not denied any relevant service. Similarly, HIV-positive individuals attending ART clinic, pregnant adolescent girls attending PMTCT clinic below 18 years of age, and those who decline finger prick collection for the BD FACSPresto study were not included in the study.

### Study design

This study investigated validity of POC BD FACSPresto^™^ CD4 analyzer for CD4+ cell count using finger prick capillary samples in comparison to a reference standard flow cytometry method, BD FACSCount using same participants venous sample. The study was designed within ART and PMTCT clinics using paired measurements of same patient sample on both BD FACSPresto and BD FACSCount system. At the ART clinic, 150 consenting patients were sent to the laboratory where venous blood sample was collected as part of routine laboratory monitoring for HIV positive patients by two trained laboratory staff. Patients who were finger pricked for BD FACSPresto testing were also bled for routine CD4 testing on BD FACSCount analyzer. The venous EDTA blood sample collection is part of routine laboratory monitoring for all HIV positive patients. Samples were drawn for routine monitoring of patient who declined participation in the BD FACSPresto study while treatment decisions for all patients were made based on the CD4 values obtained from the BD FACSCount analyzer. The BD FACSPresto^™^ analyzer result was used for research purposes only and the results were not handed over to the client nor recorded in the laboratory order and request form from the clinic. At the PMTCT clinic, 150 finger pricked capillary samples were tested on BD FACSPresto analyzer by a trained Nurse while matching samples was drawn by the Nurse using vacutainer EDTA tubes and sent to the hospital laboratory for comparison with the reference flow cytometer BD FACSCount. The health care workers (HCWs); two laboratory scientists and a PMTCT nurse, who provided CD4 testing related services to patients in the study were interviewed to assess provider acceptability.

#### Blood collection and testing on BD FACSPresto for CD4 count, CD4%

Finger-prick blood sample (capillary) was collected aseptically from fingertip using 1.8 mm depth lancet finger stick (Becton Dickinson Bioscience) and transferred to the BD FACSPresto labelled cartridge. The cartridge cap was closed and then placed on the BD FACSPresto work station outside the instrument for 18 minutes to allow incubation at room temperature. BD FACSPresto test cartridge contains in built control features to check the analyzer and reagent functionality daily. After incubation, the test-strip was removed and the cartridge was inserted into the analyser to read the result which takes about 4 minutes. The absolute CD4 count, %CD4 results are displayed on the screen and printed automatically. The print out result was stored at the respective point of testing (laboratory and PMTCT clinic) and not used for patients’ clinical management. Testing on BD FACSPresto analyzer was performed based on manufacturer instructions.

#### Blood collection and testing on BD FACSCount analyzer for CD4 count absolute and CD4% count

In the laboratory, venous blood samples collected were tested on BD FACSCount analyzer. Briefly, BD FACSCount CD4 reagents tube was brought to ambient temperature and vortexed upright for 10 seconds before it was opened for use. Fifty (50) μl of whole blood was added to the CD4 reagent tube containing CD3/CD4 PE monoclonal antibody (Becton Dickinson, USA). The tube was incubated in the dark for 30 minutes at room temperature and 50μl of fixative (5% formaldehyde in PBS) was added and vortexed before reading on Becton Dickson FACS machine according to manufacturer’s instruction using CD4 Absolute and CD4% count software [19].

#### Quality control

To ensure reproducibility and precision of CD4 count testing, daily quality controls were run for the BD FACSCount and BD FACSPresto. The BD FACS count machine quality control pack (BD Biosciences, San Jose, CA) was analyzed by running low, medium and high bead count following manufacturer’s procedures. The outcome reading of “passed control” indicated the testing process was under control such as reagents, equipment, personnel and standard operation procedures was followed before clients’ sample were tested. For BD FACSPresto, daily quality control was ensured by analyzing CD Chex Plus BC low and normal control (Streck, Omaha, NE).

#### Statistical analysis

Data was entered into Microsoft Excel and exported into STATA version 12 for analysis. The variable of interest was absolute CD4 count. Absolute CD4 count was reported as median values with accompanying interquartile range. Pearson correlation was calculated for each pair of results generated by BD FACSPresto and BD FACSCount. The accuracy of the BD FACSpresto machine in comparison to the BD FACSCount was evaluated using the Bland-Altman method, and assessed statistically with Pitman’s test of difference in variance. Sensitivity, specificity and misclassification rates of BD FACSPresto were calculated at CD4 threshold of 500 cells/μl (the 2013 eligibility threshold by WHO before the current UNAIDS Test and Start guideline) compared with results of BD FACSCount. McNemar test was performed to determine any significant differences in the misclassification between BD FACSPresto and BD FACSCount. Statistical significance was set at p-value<0.05.

#### Ethical considerations

The study protocol was reviewed and approved by FHI 360 Office of International Research Ethics (OIRE), North Carolina, IRB No 614211–1. Ethical approval was also received from Kano State Hospital Management Board, Ministry of Health with reference: HMB/GEN/488/VOL1.

## Results

A total of 300 participants were enrolled. Of these, 150 participants each were tested at the PMTCT and ART clinic respectively. The mean age of study participants tested at the PMTCT clinic was 33 years (range; 18–58) while those tested at the ART clinic was 37 years (range;18–70) (Table 1). The median CD4 count for BD FACSPresto and BD FACSCount machine for all study participants were 495.5 cells/μl (range: 304–682 cells/μl) and 462 cells/μl (range: 293.5–657 cells / μl) respectively. The Pearson correlation coefficient was 0.99 (p<0.001).

**Table 1:** Characteristics of study participants.

Variables	Total (n)	PMTCT Clinic	ART Clinic
Number of Subjects	300	150	150
Mean Age (range)	34.59(18–70)	32.66 (18–58)	36.53 (18–70)
Median CD4 count (IQR) BD FACSCount	462 (293.5–657)	456.5 (270–656)	481.5 (316–658)
Median CD4 Count (IQR) BD FACSPresto	495.5 (304–682)	446 (281–672)	495.5 (327–704)
Median CD4 Percent (IQR) BD FACSCount	21.95 (13.97–28.5)	21.7 (13.9–28.9)	21.9 (14.1–28.2)
Median CD4 Percent (IQR) BD FACSPresto	469.5 (304–682.5)	446 (281–672)	495.5 (327–704)

**Fig 1:**
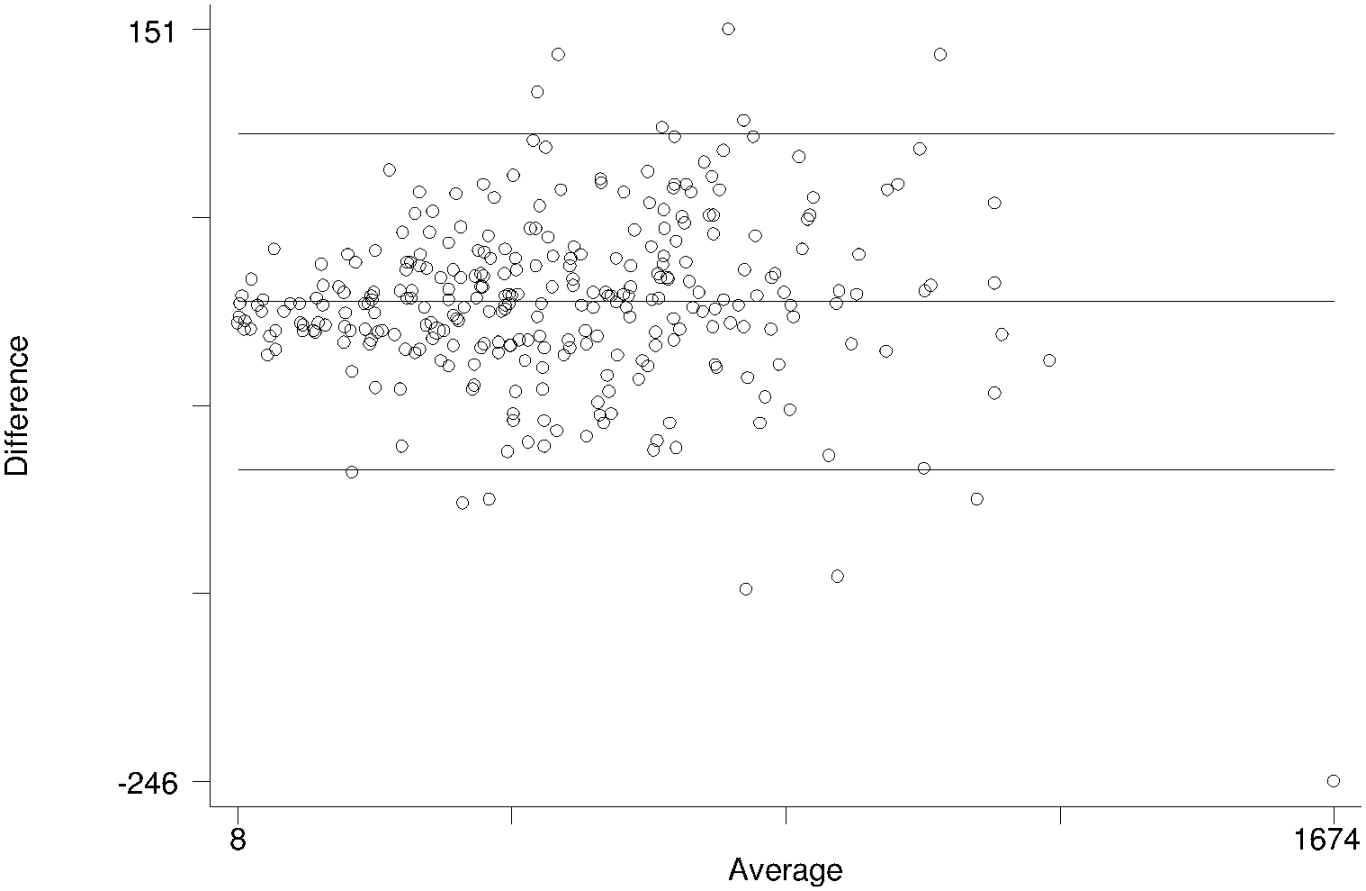
Bland-Altman plot to evaluate the difference between CD4 counts obtained using BD FACSPresto versus BD FACSCount for testing samples of all study participants.

The overall mean bias and limits of agreement (LOA) for CD4 counts between BD FACSPresto and BD FACSCount machine were 7.49 (95% CI: 2.44 to 12.54) cells/μl and -81.4 to 96.39 cells/μl, respectively. Bland–Altman analyses demonstrated close agreement between BD FACSPresto and BD FACSCount results for all study participants (Fig 1). The Pitman test confirmed a non-significant difference (P = 0.95) in the variability between BD FACSPresto and BD FACSCount results. The mean bias of the BD FACSPresto machine tested by nurses was 3.76 (95% CI: -4.13 to 11.65) cells/μl relative to the BD FACSCount, with 95% LOA from -94.03 to 101.55 cells/μl compared to 11.22 (95% CI: 4.88 to 17.56) cells/μl mean bias by laboratory scientists relative to the BD FACSCount, with 95% LOA from -67.43 to 89.87 cells/μL.

Sensitivity and specificity of BD FACSPresto at 500 cells/μl thresholds were 95.1% and 97.1% respectively, compared to BD FACSCount. The positive and negative predictive values of BD FACSPresto was 97.5% and 94.4% at 500 cells/μl. The sensitivity and specificity were similar when BD FACSPresto machine was operated by a nurse or laboratory scientist. When testing was performed by the nurse, sensitivity and specificity at 500 cells/μl thresholds were 95.2% and 96.9% respectively. When testing was performed by the laboratory scientist, sensitivity and specificity at 500 cells/μl thresholds were 94.9% and 97.2% respectively.

Using a threshold of 500 cells/μl, 5.2% (8/154) were misclassified above this threshold by BD FACSPresto compared with BD FACSCount for all study participants. Also, 3.0% (4/134) were misclassified below this threshold by BD FACSPresto compared with BD FACSCount. Hence 4.0% (12/300) were misclassified either above or below the threshold of 500 cells/μl set (Table 2). There were no statistically significant differences in the misclassification between BD FACSPresto and BD FACSCount results (p = 0.23). The misclassification rates were similar when BD FACSPresto machine was operated by a nurse or laboratory scientist. When testing was performed by the nurse or the laboratory scientist, 4.0% (6/150) were misclassified either above or below the threshold of 500 cells/μL.

**Table 2:** Two-by-two table of the BD FACSPresto compared with FACSCount using a CD4 threshold of 500 cells / μl (All study participants).

FACSCount	Total	FACSPresto ≤500	FACSPresto >500
**≤500**	162	154	8
**>500**	138	4	134
**Total**	**300**	**158**	**142**

## Discussion

Irrespective of the newly adopted WHO ART guideline and recommendation CD4+ T-lymphocyte count will remain relevant as one of the biomarkers to identify patients who are highly immunocompromised for optimal management and monitoring of HIV-infected patients on ART especially in resource limited settings. However, access and coverage remains a challenge in such environment. POC CD4 enumerating equipment provides an alternative to standard equipment and makes CD4 count readily available at remote locations and lower level of health facilities thereby increasing access and coverage of quality of ART services with considerable improvement in patients' management.

The findings above revealed close comparison between data obtained from BD FACSPresto when compared with the reference method, BD FACSCount flow cytometry and this is similar to findings reported elsewhere [13, 17, 18]. Evidence from literature supports that POC CD4 testing has shown promising results in improving linkages to ART care [11, 16, 20] and importantly POC testing, also known as near patient testing, offers significant potential advantages over laboratory-based testing [21].

Our Bland–Altman analysis demonstrated close agreement between BD FACSPresto and BD FACSCount results for all study participants with no statistical significant difference (p = 0.95) in the variability between results from both equipment. BD FACSPresto had high sensitivity and specificity and lower misclassification value of 3.0%. The sensitivity and specificity were similar when BD FACSPresto machine was operated by a nurse or laboratory scientist. This attests to the fact that BD FACSPresto can accurately identify those above the threshold of 500 cells/ μl irrespective of location or operator. Similar observation had been reported in Kenya [18]. The 5.2% misclassification above threshold found in our study represents a possible mis-categorization as healthy, some patients who should have been otherwise prioritized for ART and possible check for opportunistic infection (cryptococcal) using the ART guidelines in place before 2016. The current treatment guideline however recommends for all to be treated irrespective of the CD4 count.

The positive and negative predictive values at threshold of 500 cells/μl were more than 90% in the study and similar to what was report in another study [13] making the BD FACSPresto a reliable POC system to provide accurate CD4 results. Furthermore, a close agreement was observed between BD FACSPresto and BD FACSCount results of patients tested by a nurse with no significant difference in variability (P = 0.4). A similar study in Ugandan reported high correlation between PIMA and BD FACSCalibur [20] and another study in Zimbabwe [14] observed no significant difference in mean absolute CD4 either by a nurse or a laboratory technician. Analysis using Pitman test confirmed a non-significant difference (p = 0.18) in the variability between BD FACSPresto and BD FACSCount results of ART patients tested by the two independent laboratory scientists. The improved performance of BD FACSPresto POC CD4 analyzer at the PMTCT clinic could be due to the fact that the nurse in our study received a one-day training prior to the study. This is however expected to be standard service delivery practice—training of healthworkers before handling of equipment. POC CD4 testing when placed at outside laboratory can improve access to treatment and quality of care provided by enabling decentralization and reduction in patient’s attrition. Although multiple factors are responsible for loss to follow up, availability of CD4 testing at point of care will help in reducing the number of clinic visits, thus helping in reducing loss to follow up. A recent report from Africa showed that POC CD4 testing could successfully reduce the pre-treatment loss to follow-up [9].

BD FACSPresto analyzer has the advantage of having the complete incubation process during testing take place outside the machine and thus the sample throughput is relatively high and could take more samples in a medium yield site. The analyzer also provides percentage of CD4+ cells and hemoglobin level; hence, it would be useful for monitoring the paediatric population and anaemic condition in pregnancy. The analyzer can be used after minimum training and can be run by the clinical staff such as nurses where multi-tasking may be necessary due to shortage of trained laboratory staff. The operators also described that the analyzer is easy to use, the use of lancet for capillary blood has a higher risk of repeat pricks causing discomfort but patient waiting time is reduced overall.

### Limitations

This study presents with some limitations, firstly, due to sample size and study design, the current study does not provide a clear assessment of patients with extreme CD4. The population for this validation were HIV infected adults because HIV infected pediatric clients were not included in the validation.

## Recommendations

CD4 will continue, in the immediate period, to play a significant role in resource limited places where viral load testing capacity is not readily available. It will continue to have a long term role in patient monitoring for various clinical decision including treatment prioritization, risk of opportunistic infections and assessing risk of ARVs hypersensitivity [22]. Hence the need for integration of POC CD4 testing into the ART/PMTCT program. Based on the settings in rural Nigeria, there is the need for the consideration to pilot the use of equipment like the FACSPresto CD4 count within the community to improve services provided to clients. There is need to properly train all non-laboratory health care worker on stick capillary sample for BD FACSPresto CD4 count to avoid wastages, repeat of procedure and loss of patient confidence in a non-laboratory setting. The pattern and variability of performance between different POC equipment of point of care testing such as PIMA against BD FACSPresto point of care testing needs evaluation to determine both POC comparability in our setting.

## Conclusions

Overall, BD FACSPresto POC CD4+ count finger stick capillary testing produces results which are reliable in comparison to venous sample cytometry method and had no significant difference in variability observed between laboratory personnel and non-laboratory operators. It will be a valuable instrument in increasing access to and coverage of CD4 testing in developing countries. The introduction of the BD FACSPresto POC has a high potential in reducing patients waiting time and improving the overall quality of ART service and clients’ satisfaction especially in rural settings.

## Supporting information

S1 File BD FACSPresto users perspectives. (XLSX)
